# Diagnosis of COVID-19 in children: the story evolves

**DOI:** 10.1186/s12916-020-01631-9

**Published:** 2020-05-28

**Authors:** R. Harwood, I. Sinha

**Affiliations:** 1 Alder Hey Children’s NHS Foundation Trust, Liverpool, UK; 2 University of Liverpool, Liverpool, UK

**Keywords:** COVID-19, SARS-CoV-2, Children, Presenting features, Diagnostic tests, Computed tomography

## Background

Our understanding of all aspects of coronavirus-19 (COVID-19) is evolving. Some aspects already appear clear: children are significantly less likely to be diagnosed with SARS-CoV-2 than adults [1] and those who are have less likelihood of being severely affected [2–4]. Nevertheless, significant concerns remain about the small number of children reported as requiring ventilatory support and who have died as a result of COVID-19 [2].

## Clinical features of SARS-CoV-2 in children

Details about SARS-CoV-2 infection in children first emerged in small, retrospective case series from China. Children in these series form a relatively heterogenous population, with up to 40% being asymptomatic. These early studies identified that children who displayed symptoms were less likely to have a cough and fever, but more likely to have vomiting or diarrhoea compared to adults and were also likely to present to hospital earlier in the disease. These findings are borne out in larger case series from China, Spain and the USA where fever is reported in 40–60% of children, cough in 19–65% of children and diarrhoea and vomiting in approximately 10% of children diagnosed with SARS-CoV-2 infection when the number of asymptomatic carriers included in the study are low [2, 5–7].

Predictive markers of severe SARS-CoV-2 infection are not yet fully understood, with ongoing evaluation of the effects of gender, ethnicity, age, co-morbidities and biochemical and radiographic features on outcome. Much of this evaluation will be undertaken in adults, but its applicability to children, particularly younger children and infants, has questionable relevance. In the published literature, there do not appear to be specific co-morbidities which increase the risk of requiring hospitalisation for SARS-CoV2 infection. A small Chinese study describes 46% of hospitalised children having cardiac co-morbidities, none of whom required intensive care support and were successfully discharged. Two children with underlying congenital heart disease required mechanical ventilation and one of these children also required haemofiltration. The USA reports that 7% of children diagnosed with COVID-19 and with details entered into the CDC database have cardiovascular disease and up to 12% having chronic lung disease, including asthma [2].

There is good evidence in adult patients that lymphopaenia is a good predictor of disease severity for COVID-19. The paediatric studies which report blood results have small numbers of patients with a variety of phenotypes within them (asymptomatic, mildly, moderately and severely symptomatic), making it difficult to draw conclusions about the findings [5]. However, lymphocytes are reported as being low, normal and high in COVID-19-positive patients, whether asymptomatic or not. CRP and procalcitonin appear to be mildly elevated, particularly in children with features of pneumonia. Liver enzymes are increased in up to 15% of children diagnosed with COVID-19, primarily in symptomatic children (< 1% of asymptomatic children had elevated alanine aminotransferase). D-Dimer is more likely to be elevated in symptomatic children infected by SARS-CoV-2, being documented in up to 18% of children.

Computed tomography (CT) has been used extensively in China as a diagnostic tool for SARS-CoV-2. Reverse transcriptase polymerase chain reaction (RT-PCR) has been reported as having a false negative rate up to 30% [8], prompting clinicians to look at alternative diagnostic modalities. One hundred fifty-eight Chinese children who had been in contact with COVID-19 had RT-PCR testing and CT chest [9]. Notably, 3% of children were initially negative on RT-PCR testing but positive on a subsequent swab, whilst 4% of children were positive but asymptomatic with normal CT findings. These potentially false negative and positive rates are in-keeping with recent literature [10].

Importantly, the article highlights CT abnormalities which can be found in the majority of children with confirmed COVID-19. Ground-glass opacity, local and bilateral patchy shadowing are the three most common findings and are reported in 36–100% of children with confirmed SARS-CoV-2 in other series [5, 6]. CT abnormalities were significantly more likely to be found in children with confirmed COVID-19, but nevertheless, 25% of children without confirmed COVID-19 on RT-PCR had CT changes too, with a higher proportion of these children having mycoplasma pneumonia. Many of the studies reporting CT changes associated with COVID-19 have high rates of concomitant infection including mycoplasma and influenza B which may account for this discrepancy and makes CT a poor diagnostic modality.

The need for admission to the intensive care unit (ICU) and the mortality rate of children with COVID-19 are unclear. At least 7/246 (3%) Chinese children have been reported as requiring intensive support with 1 death reported [5]. The rates of ICU admission internationally may be higher, with a Spanish series reporting almost a 10% requirement for ICU support but no deaths [7], and similarly, in the USA, 15/147 (10%) of children required ICU support and 3 deaths have been reported [2].

Minimal data are published on the children who are affected by severe SARS-CoV-2. These small numbers make it difficult to draw conclusions about which children are at risk from severe complications of SARS-CoV-2 infection and death. However, the overall message about COVID-19 in children is reassuring. Infection rates of SARS-CoV-2 in children are lower than those in the general population. The majority of infected children are asymptomatic or have mild disease, and do not require invasive support. There is a suggestion that children with cardiac co-morbidities may be at higher risk of developing severe COVID-19. This is based on a limited number of small case series and incomplete reporting into CDC database which may reveal a reporting bias.

## Conclusions

The presenting features of COVID-19 differ in children from adults, with lower rates of fever and cough and more prominence of vomiting and diarrhoea. Currently, haematological, biochemical and radiological findings in children infected with COVID-19 are not diagnostic and do not consistently predict severe disease.

Prospective research is required to help identify any patient groups who are at risk of developing severe infection and features which may be used to identify these children early in the disease course.

## Data Availability

Not applicable

## Abbreviations

CDC: Centre for Disease Control
COVID-19: Coronavirus-19
CRP: C-reactive protein
CT: Computed tomography
ICU: Intensive care unit
RT-PCR: Reverse transcriptase polymerase chain reaction
USA: United States of America
